# Effects of sponge-derived Ageladine A on the photosynthesis of different microalgal species and strains

**DOI:** 10.1371/journal.pone.0244095

**Published:** 2020-12-31

**Authors:** Carolin Peter, Silke Thoms, Florian Koch, Franz Josef Sartoris, Ulf Bickmeyer

**Affiliations:** 1 Division of Biosciences, Department of Ecological Chemistry, Alfred Wegener Institute Helmholtz Centre for Polar and Marine Research, Bremerhaven, Germany; 2 Department 2, University of Applied Sciences, Bremerhaven, Germany; 3 Division of Biosciences, Department of Integrative Ecophysiology, Alfred Wegener Institute Helmholtz Centre for Polar and Marine Research, Bremerhaven, Germany; Pondicherry University, INDIA

## Abstract

Fluorescent natural compounds have been identified in several marine hosts of microalgae. Their prevalence, and the energy the host is expending on their synthesis, suggests an important, yet poorly understood ecological role. It has been suggested that some of these natural products may enhance the photosynthesis of microbial symbionts. In this study, the effect of Ageladine A (Ag A), a pH-dependent fluorophore found in sponges of the genus *Agelas*, on the photosynthesis of nine microalgal species and strains was examined. The data showed that the variety of effects of Ag A additions differed between species, and even strains within a species. While in one strain of *Synechococcus* sp., the presence of Ag A increased gross photosynthesis under UV light exposure, it decreased in another. And while in the chlorophyte *T*. *chuii* overall metabolic activity was greatly reduced under all forms of lighting, photosynthesis in *T*. *lutea* was positively affected by the addition of Ag A. The variety of effects of Ag A on photosynthesis observed in this study indicate a complex interaction of Ag A with microalgal cells and suggests that a host may be able to shape its own symbiotic microbiome with self-produced natural products.

## Introduction

The pH-dependent fluorophore Ageladine A (Ag A) was discovered in 2003 in lipophilic extracts of the marine sponge *Agelas nakamurai* [1]. While at neutral and alkaline pH the uncharged species dominates, single and double protonated states of Ag A are formed when the pH decreases. This shift in molecular composition corresponds to an increase in fluorescence, which peaks at pH 3–4. The peak in excitation wavelength in water at 370 nm corresponds to an emission ranging from 415 nm to 500 nm with a maximum at 415 nm [2], however these excitation/emission profiles can change with chnaging environments [3].

Ag A is a known angiogenesis, matrixmetalloproteinase [1] and kinase inhibitor [4]. But its specific function within the sponge holobiont has not yet been identified.

After Read et al. [5] discovered fluorophores in demosponges which, when excited by ultraviolet/blue light emit blue to green light, Schlichter et al. [6,7] reported a similar chromatophore system in the deep-water coral *Leptoseris fragilis*. Since this coral harbours zooxanthellae (*Symbiodinium microadriaticum*), it was hypothesised, that the products produced by the host may play an important role in the photosynthetic efficiency of its symbionts. It was proposed that only the combination of low light adapted endosymbionts and a light-amplifying mechanism by the host allowed phototrophic growth at 95–145 m, the depth where *L*. *fragilis* is typically found [8].

Given the fluorescent characteristics of Ag A and the prevalence of phototrophic symbionts in sponges [9–15], this could also be the role of Ag A in the sponge holobiont.

Due to its molecular structure, Ag A enters cells and cellular compartments at a neutral pH. Once inside an acidic compartment, however, it becomes protonated and trapped. Based on this, Bickmeyer et al. [16] hypothesised that Ag A accumulates in the thylakoid lumen where it provides light to algal pigments, thus increasing photosynthetic efficiency under UV radiation. Indeed, their experiments with *Synechococcus bacillaris* showed that photosynthetic oxygen evolution increased 2.5-fold after the incubation with Ag A.

Another effect of Ag A stems from its direct interaction with certain photosynthetic pigments. Peter et al. [17] reported significant effects of Ag A on the fluorescence spectra of microalgae indicating direct or indirect interactions between Ag A and algal pigments such as phycoerythrin and chlorophyll *a* (chl *a*). Thus, it is likely that the photosynthetic efficiency of algae possessing these pigments as main light harvesting molecules may be impacted.

Many sponges harbour photosynthetic symbionts belonging to diverse phyla, such as cryptophytes, diatoms, dinoflagellates, and members of the cyanobacterial genus *Synechococcus* [9–15]. Thus the starting hypothesis of this study was, that the effect of Ag A on photosynthesis under UV radiation may be universal. The magnitude of this effect and its relevance when algae are offered a wider spectrum of light, however, may be governed by the various of pigment profiles produced by the different algal phyla.

The results presented here highlight a complex and diverse reaction of different algae to Ag A additions.

## Material and methods

In this study, four prokaryote strains and five eukaryote species spanning three algal phyla were examined. Four cyanobacteria strains belonging to two pigment types [18] differing in the composition of their phycobilisomes (S1 Table) were used. While *Synechococcus* sp. RCC1084 and *S*. *bacillaris* CCMP1333 belong to the green pigment type 1, *Synechococcus* sp. RCC539 and RCC791 belong to the orange pigment type 3. The five eukaryotes included the three chlorophytes *Chlorogonium elongatum* (by courtesy of B. Seah), *Micrasterias americana* (SAG 151.80) and *Tetraselmis chuii* (SAG 8 6), the haptophyte *Tisochrysis lutea (CCAP 927/14)* and the cryptophyte *Rhodomonas salina* (K-1487).

### Culture conditions

The marine algae and freshwater algae were cultivated in K-medium [19] and DY-Vm medium (Bigelow Laboratory, National Centre for Marine Algae and Microbiota, East Boothbay, ME, USA), respectively, made from autoclaved, 0.2 μm North Sea water (salinity 34 psu) and 0.1 μm ultrapure water. All stock cultures were grown in duplicate cell culture flasks (T25, Sarstedt, Nümbrecht, Germany and T75, TPP Techno Plastic Products AG, Trasadingen, Switzerland).

The eukaryotes were cultivated at room temperature (21 ± 1°C) with a 18:6 h light:dark cycle of 70–80 μmol photons m^-2^ s^-1^. The *T*. *chuii* and *T*. *lutea* cultures were kept on a shaker (KS 130 control, IKA-Werke GmbH & Co. KG, Staufen, Germany) at 100 rpm. The *Synechococcus* cultures were kept under continuous light in a MIR-253 Sanyo incubator (LabX, Midland, ON, Canada) at 25°C and 30 μmol photons m^-2^ s^-1^. The light intensities were measured using a spherical underwater quantum sensor (SPQA) connected to a LI-1000 DataLogger (LI-COR Biosciences GmbH, Bad Homburg, Germany).

### Oxygen evolution experiments

The oxygen evolution experiments were performed with cultures in exponential growth and consisted of a four-hour incubation period under the respective growth conditions. The chemicals used were dimethyl sulfoxide (DMSO, Carl Roth GmbH + Co. KG, Lichtentanne, Germany), 6 mM ageladine A (Ag A, Marnas Biochemicals GmbH, Bremerhaven, Germany) dissolved in DMSO and 0.5 M sodium bicarbonate (Sigma Aldrich, St. Louis, MO, USA). The concentration of Ag A used was chosen according to the species’ or strain’s tolerance to the substance as determined in preliminary experiments. For *Synechococcus* sp. RCC1084, *C*. *elongatum*, *R*. *salina* and *T*. *lutea*, a final concentration of 5 μM Ag A was used. For *Synechococcus* sp. RCC539, RCC791, *S*. *bacillaris* CCMP1333 and *M*. *americana* the final concentration was 10 μM Ag A. *T*. *chuii* was incubated with 30 μM Ag A. In order to eliminate potential effects of Ag A´s solvent DMSO on the cells or the fluorescence signal, controls were also incubated with 0.5% vol DMSO. The final concentration of sodium bicarbonate in all treatments was 2.5 mM.

Five samples of 2mL culture were prepared in parallel in UV-grade cuvettes. Oxygen concentration within the algae samples was measured before and after an exposure to the respective irradiance for a period of 90 min using oxygen micro-optodes (DP PSt3 L8 STO NOP; PreSens Precision Sensing GmbH, Regensburg, Germany) and expressed as percent saturation, based on a calibration using sodium thiosulfate (0% O_2_) and saturated water (100% O_2_).

**Fig 1:**
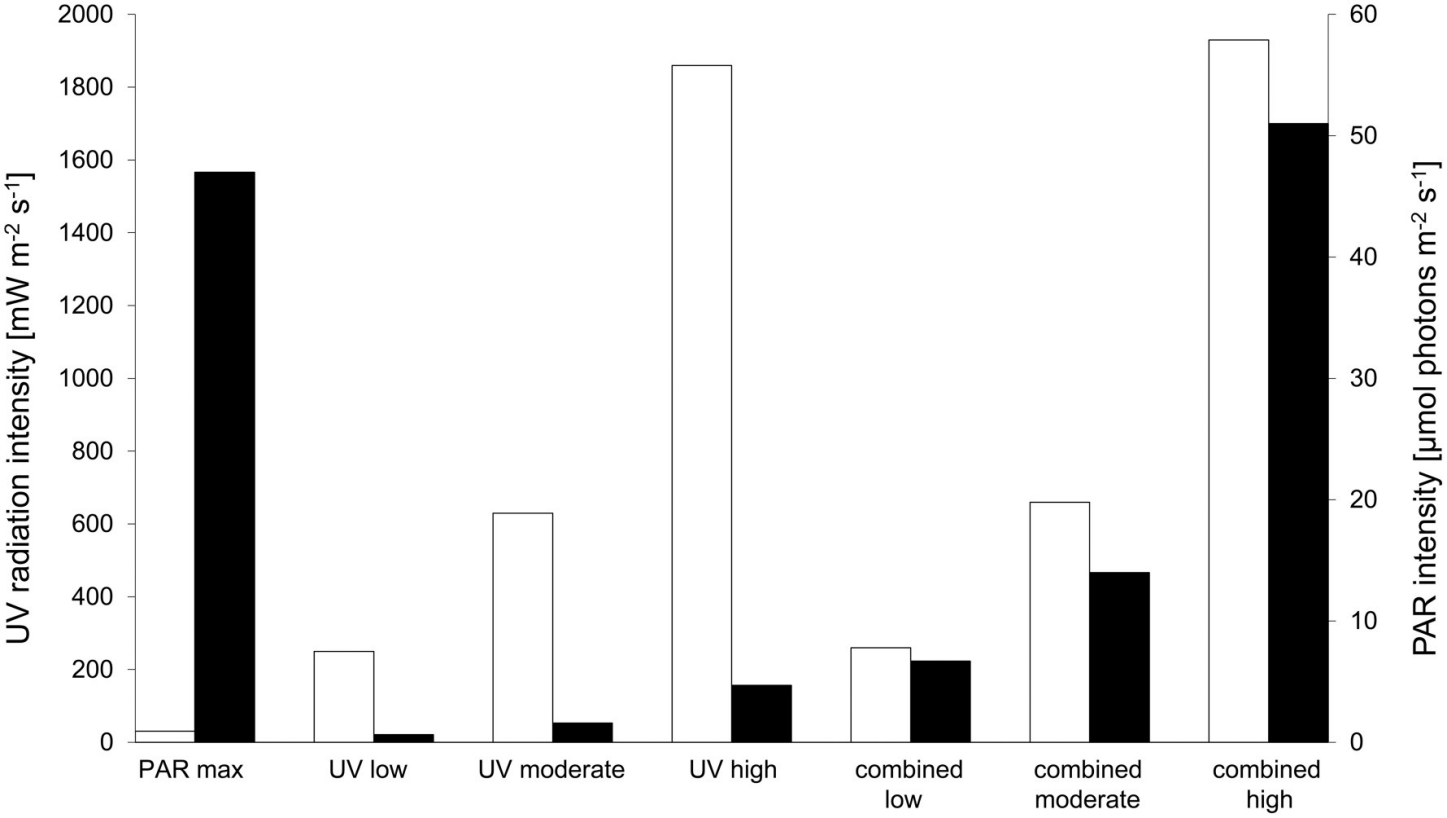
Experimental light conditions displayed as a combination of UV radiation (white bars in mW m^-2^ s^-1^) and PAR (black bars in μmol photons m^-2^ s^-1^). More detailed values can be found in S2 Table.

During the experiments, the samples were illuminated by an UVA-340 (Q-Lab Corporation, Cleveland OH, USA) and a Lumilux De Luxe L 36W 954 daylight lamp (Osram, Munich, Germany). The UV radiation intensities were measured with a Ramses ARC system (TriOS Mess- und Datentechnik GmbH, Rastede, Germany) while the PAR intensities were measured with a spherical underwater quantum sensor (SPQA) connected to a LI-1000 DataLogger (LI-COR Biosciences GmbH, Bad Homburg, Germany). In the “moderate” and “low” light treatments (Fig 1) neutral density screening was used, reducing the light intensity without affecting its spectral composition.

Cell densities were assessed at the start and end of the experiments. In *C*. *elongatum* and *M*. *americana* cell densities were determined by optical microscopic counts with a gridded Sedgewick Rafter cell S50 (Pyser–SGI limited, Kent, UK) under an Axiovert 25 inverted light microscope (Carl Zeiss AG, Oberkochen, Germany). *T*. *chuii*, *R*. *salina*, and *T*. *lutea* were analysed by a Beckman Coulter Multisizer^™^ 3 Coulter Counter^®^ (Beckman Coulter GmbH, Krefeld, Germany) and the four *Synechococcus* strains were analysed by flowcytometry (BD Accuri c6; Becton, Dickinson and Company, Franklin Lakes, NJ, USA).

### Data analysis

The data were analysed in Microsoft Excel (2019) and SigmaPlot version 14.0. Statistical tests used were the one sample t-test, the Student´s t-test and ANOVA based on Holm-Sidak tests. Results with p<0.05 are referred to as ‘significant’ and results with p<0.01 as ‘statistically highly significant’ from now on. All oxygen evolution data were normalised to 10^6^ cells mL^-1^ for prokaryotes and 10^3^ cells mL^-1^ for eukaryotes. All analysed raw data are enclosed in the supplementary data (S3–S11 Tables).

Gross oxygen evolution was calculated based on measured oxygen evolution and oxygen loss in darkness (S12 and S13 Tables).

## Results

### Oxygen evolution under pure UV radiation

The effect of Ag A differed greatly, when the algae were subjected to different intensities of UV radiation, not only between species but even between strains.

**Fig 2:**
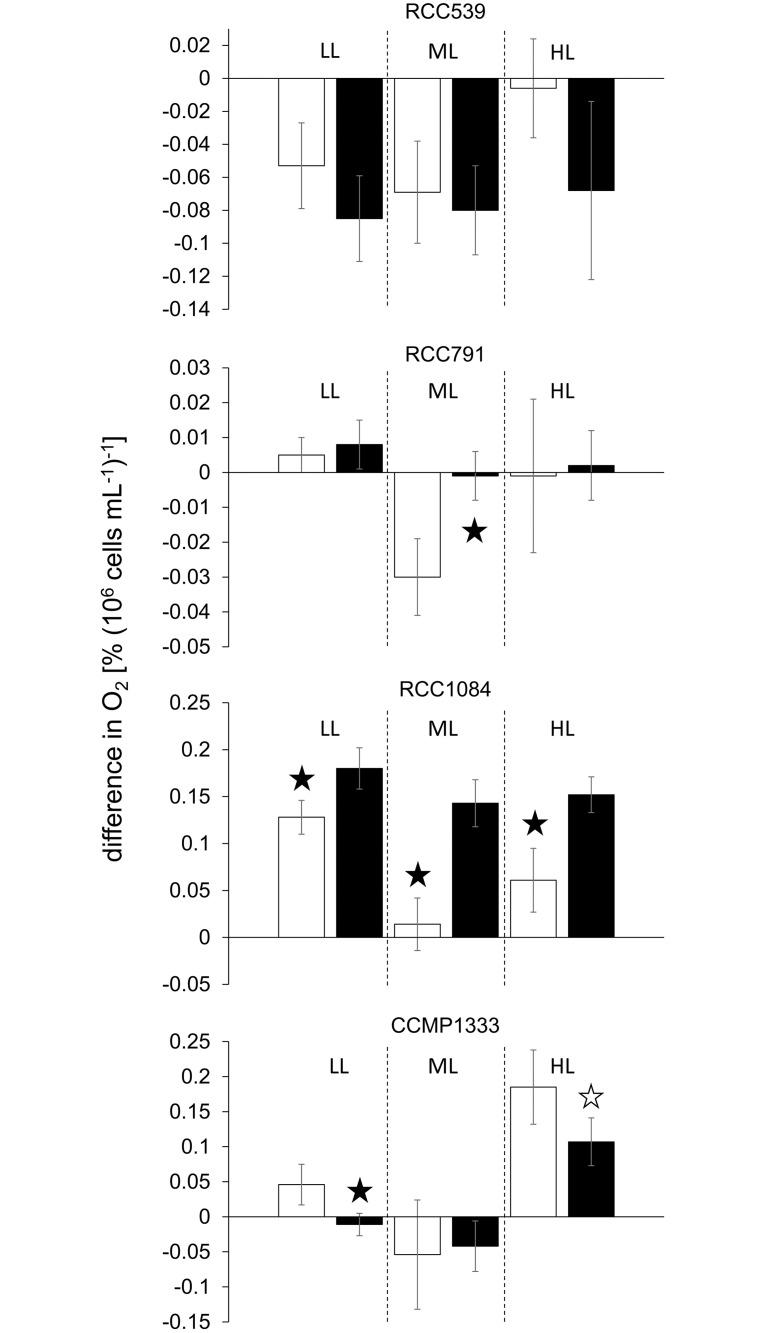
Difference in % O_2_ saturation per 10^6^ cells mL^-1^ under different UV radiation intensities as detailed in S2 Table; LL, ML and HL refer to low light, moderate light and high light conditions, respectively; values denote mean ± standard deviation (n = 5); the white and black bars denote the control and the Ag A treatment, respectively; black stars denote highly significant (p<0.01) differences between the control and the Ag A treatment, white stars denote significant (p<0.05) differences.

While *Synechococcus* sp. RCC539 showed no effect of Ag A at any UV radiation intensity, RCC1084 exhibited highly significant positive effects under all intensities (Fig 2). The increase in oxygen evolution of Ag A incubated cells compared to the control ranged from +41% at minimal intensity to +921% at moderate intensity. In *Synechococcus* sp. RCC791 and *S*. *bacillaris* CCMP1333, the effect on oxygen evolution depended on radiation intensity. While Ag A decreased the loss of oxygen in RCC791 significantly at moderate radiation intensity, it had no effect either at low or high intensity. In the case of *S*. *bacillaris* CCMP1333 on the other hand, in presence of Ag A oxygen evolution was decreased significantly by -124% and -42% at low and high radiation intensities, respectively, but remained unaffected at moderate intensity.

**Fig 3:**
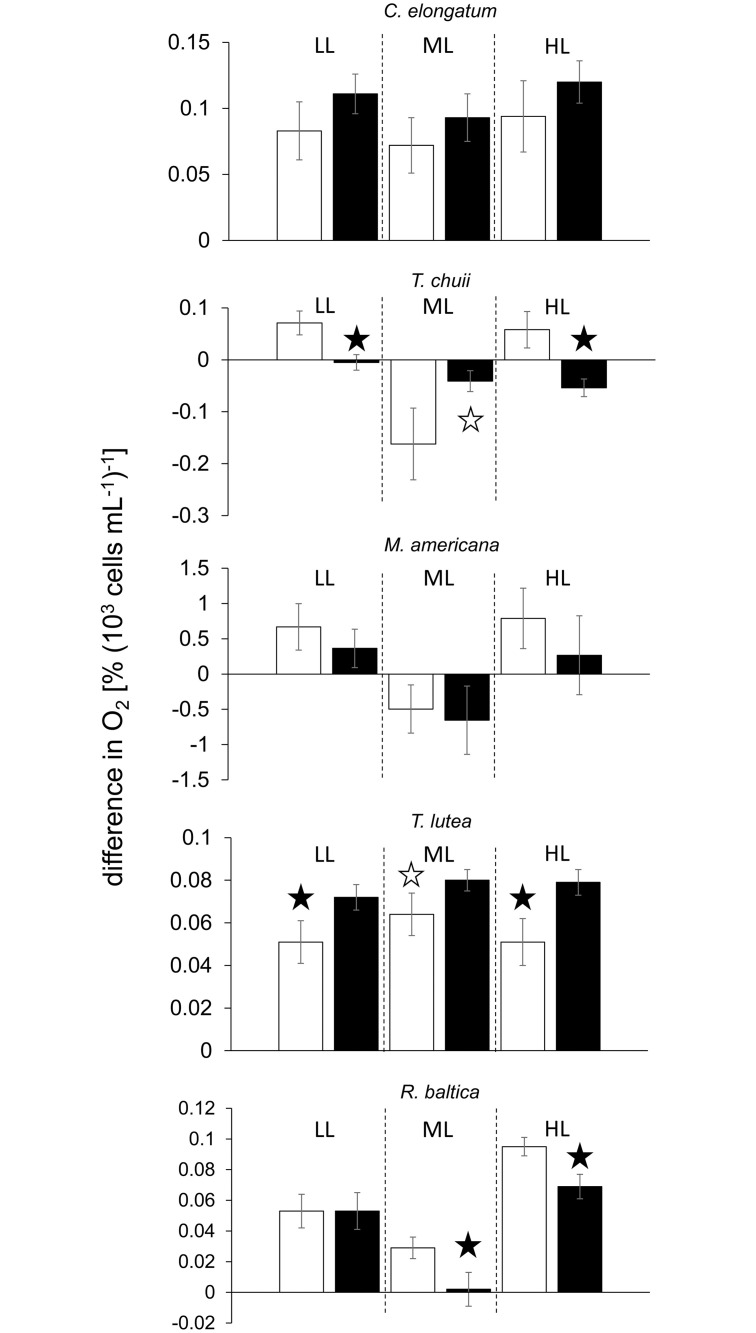
Difference in % O_2_ saturation per 10^3^ cells mL^-1^ under different UV radiation intensities as detailed in S2 Table; LL, ML and HL refer to low light, moderate light and high light conditions, respectively; values denote mean ± standard deviation (n = 5); the white and black bars denote the control and the Ag A treatment, respectively; the white and black bars denote the control and the Ag A treatment, respectively; black stars denote highly significant (p<0.01) differences between the control and the Ag A treatment, white stars denote significant (p<0.05) differences.

Of the eukaryotes examined, *C*. *elongatum* and *M*. *americana*–the two freshwater chlorophytes–behaved alike and showed no significant effect of the Ag A addition at any radiation intensity (Fig 3). Conversely, *T*. *chuii* and *R*. *baltica* did exhibit radiation intensity dependent negative effects. In *T*. *chuii*, oxygen evolution was reduced by more than 100% at low and high radiation intensity. At moderate intensity, the presence of Ag A resulted in 75% lower oxygen loss compared to the control. Even though in *R*. *baltica*, Ag A had no significant effect at low radiation intensity, at moderate light intensity a -93% decrease in oxygen evolution was observed, and at high light intensity oxygen evolution decreased by -27%. The only case, where Ag A resulted in positive effects, was in *T*. *lutea*, where it increased the oxygen evolution significantly by +41%, +25% and +55% at low, moderate and high radiation intensities, respectively.

### Oxygen evolution under a combination of UV radiation and PAR

**Fig 4:**
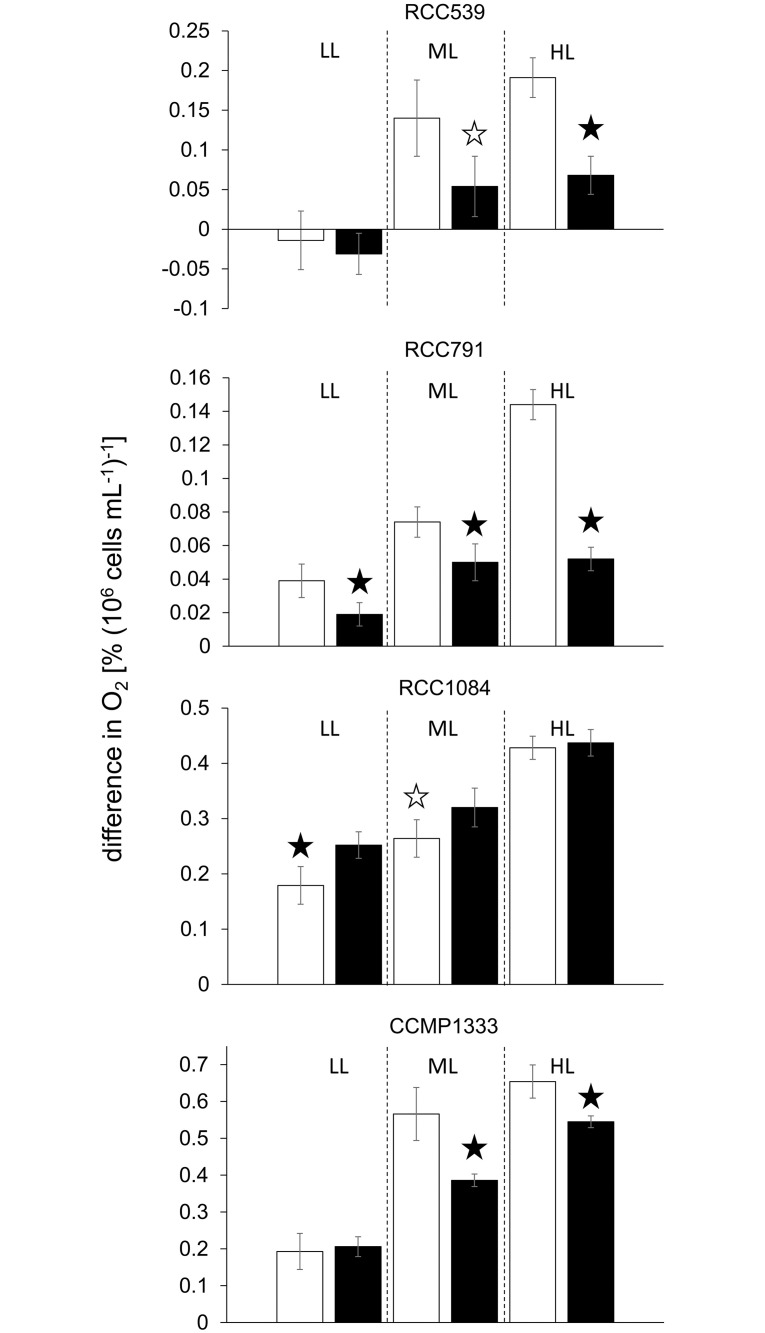
Difference in % O_2_ saturation per 10^6^ cells mL^-1^ under combinations of PAR and UV radiation at different intensities as detailed in S2 Table; LL, ML and HL refer to low light, moderate light and high light conditions, respectively; values denote mean ± standard deviation (n = 5); the white and black bars denote the control and the Ag A treatment, respectively; the white and black bars denote the control and the Ag A treatment, respectively; black stars denote highly significant (p<0.01) differences between the control and the Ag A treatment, white stars denote significant (p<0.05) differences.

When subjected to a combination of UV radiation and PAR, only the *Synechococcus* strain RCC1084 exhibited increased oxygen evolution in the presence of Ag A (Fig 4) increasing by +21% at moderate and by +41% at low intensities, but remaining unaffected at the high radiation intensity. In contrast, the other three *Synechococcus* strains tested responded to Ag A by reducing oxygen evolution (Fig 4). In RCC791, the incubation with Ag A resulted in significantly reduced oxygen evolution at all radiation intensities, ranging from -32% at moderate intensity to -64% at high intensity. In RCC539 and *S*. *bacillaris* CCMP1333, oxygen evolution at low intensity remained unaffected, but was reduced by -61% and -64% and by -32% and -17% at moderate and high intensities, respectively.

**Fig 5:**
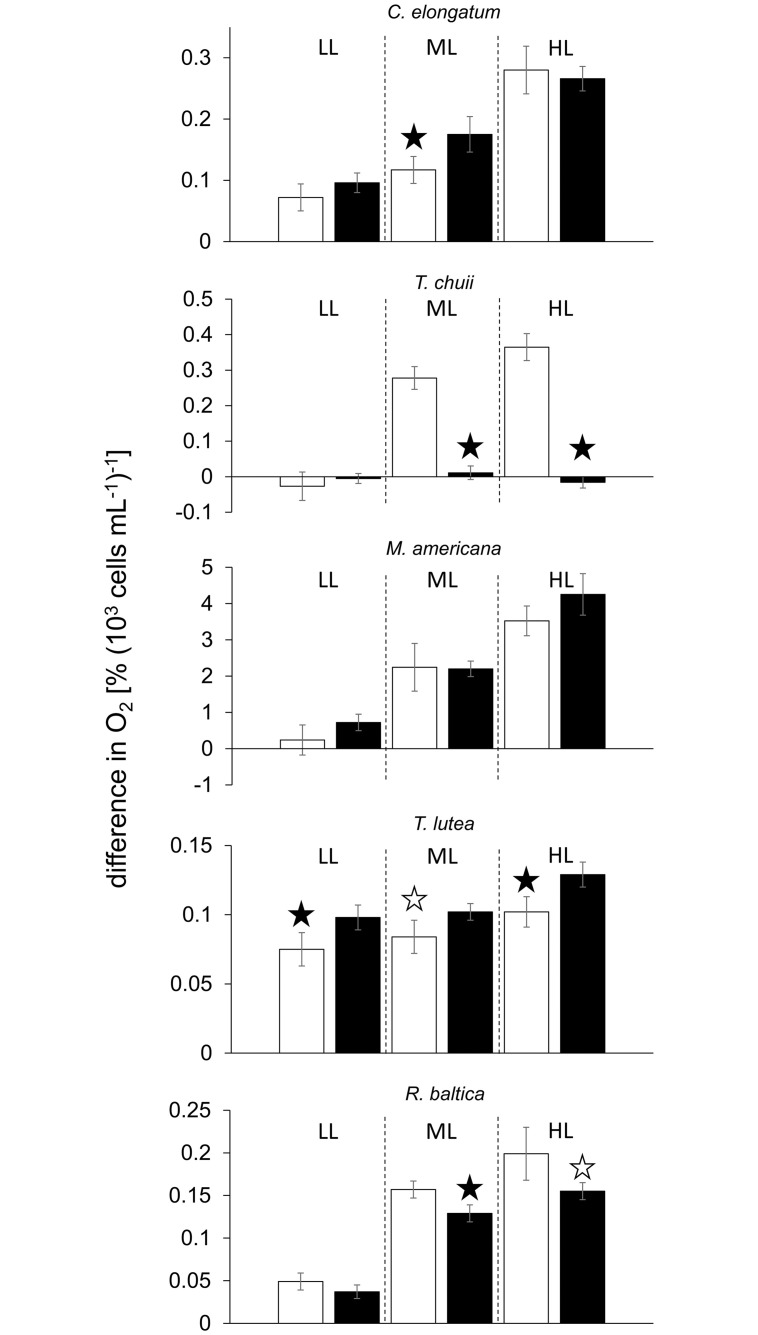
Difference in % O_2_ saturation per 10^3^ cells mL^-1^ under combinations of PAR and UV radiation at different intensities as detailed in S2 Table; LL, ML and HL refer to low light, moderate light and high light conditions, respectively; values denote mean ± standard deviation (n = 5); the white and black bars denote the control and the Ag A treatment, respectively; the white and black bars denote the control and the Ag A treatment, respectively; black stars denote highly significant (p<0.01) differences between the control and the Ag A treatment, white stars denote significant (p<0.05) differences.

In the eukaryotic species investigated, the effect of Ag A on gross photosynthetic oxygen evolution varied (Fig 5). While *M*. *americana* exhibited no significant effect of Ag A at any radiation intensity, in *T*. *lutea* oxygen evolution increased by +31% at low intensity, +21% at moderate and +26% at high intensities compared to the controls. In *T*. *chuii* and *R*. *baltica*, Ag A had no effect at low radiation intensity and affected oxygen evolution adversely at moderate and high intensity. This effect was at -96% and -104%, respectively, greater in *T*. *chuii* than in *R*. *baltica* with -18% and -22%, respectively. In *C*. *elongatum*, a significant increase in oxygen evolution in presence of Ag A was only observed under moderate radiation intensity.

### Oxygen evolution under pure PAR

**Fig 6:**
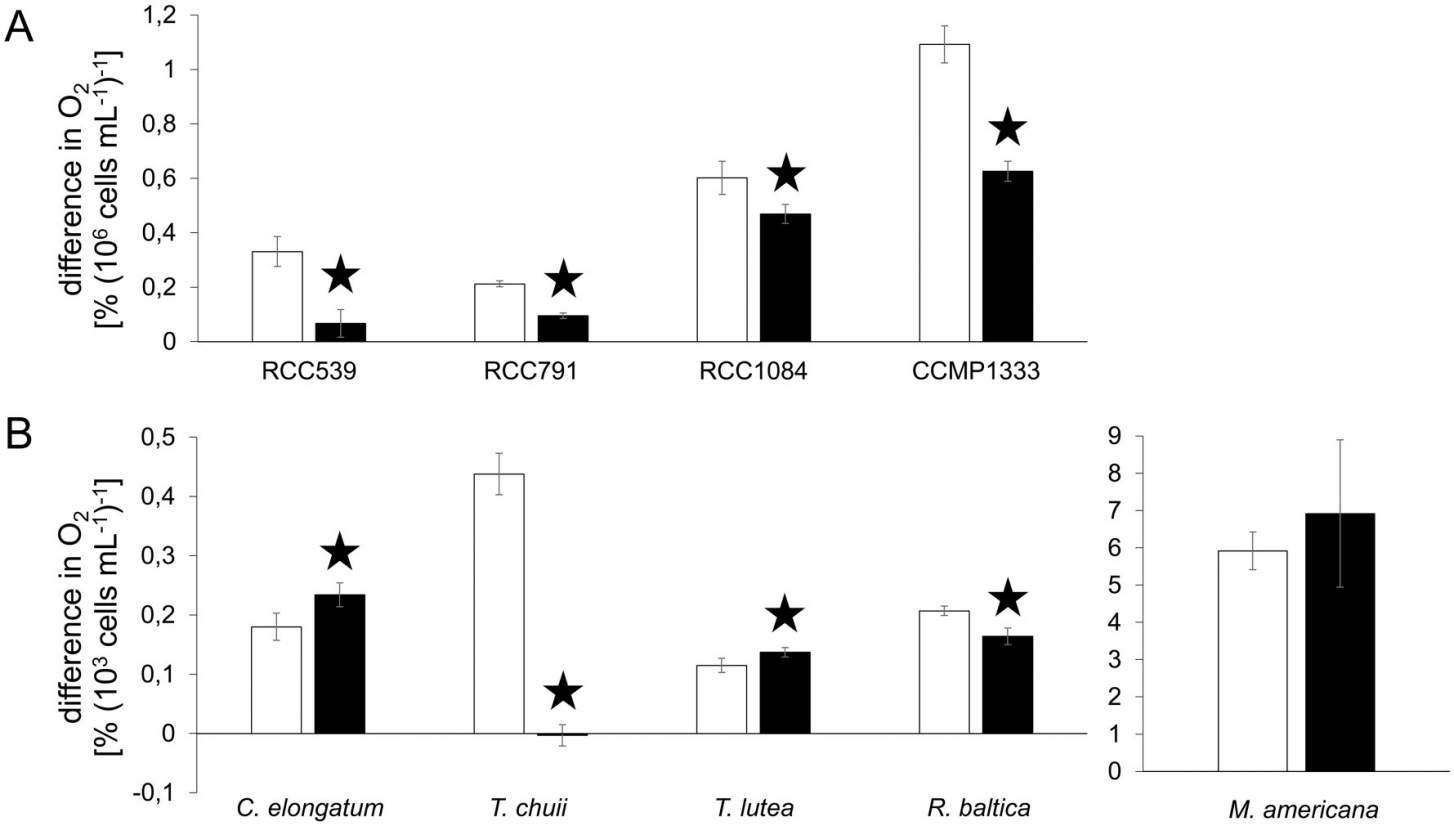
Difference in % O_2_ saturation under light treatment PAR max; values denote mean ± standard deviation (n = 5); the white and black bars denote the control and the Ag A treatment, respectively, A: *Synechococcus* strains RCC539, RCC791, RCC1084 and CCMP1333 B: *C*. *elongatum*, *T*. *chuii*, *T*. *lutea*, *R*. *baltica*, *M*. *americana*; black stars denote highly significant (p<0.01) differences between the control and the Ag A treatment.

For the four *Synechococcus* strains, the effect of Ag A under pure PAR was the same (Fig 6). The oxygen evolution was in all cases highly significantly reduced with a maximal reduction of -80% in RCC539. Of the eukaryotes, *T*. *chuii* and *R*. *baltica* showed a reduction in oxygen evolution by -101% and -21%, respectively. In *C*. *elongatum* and *T*. *lutea*, on the other hand, oxygen evolution was increased by +30% and +19%, respectively. In *M*. *americana*, no significant effect was observed.

## Discussion

### Effects of Ag A: A wavelength-transforming system

Bickmeyer et al. [16] hypothesised that UV radiation absorbed by Ag A and subsequently emitted as fluorescence in the visible spectrum could aid algal photosynthesis in *Synechococcus bacillaris*. The oxygen evolution in *S*. *bacillaris* samples incubated with Ag A increased 2.5-fold compared to the control when exposed to UV radiation (370–380 nm). Since the study also reported unaffected respiration rates, it suggested a significant increase in algal photosynthetic efficiency due to the presence of Ag A.

Since sponges have been found to harbour a plethora of microalgal species as symbionts [9–15] and molecules changing light availability in favour of photosynthesis have been found in several hosts [1,5–8], Bickmeyer et al. [16] hypothesised, that this underlying principle may be universally attributed to phototrophs.

In this case, the mechanism of the host offering a substance, which converts unusable UV radiation into radiation of wavelengths the algae can use for photosynthesis, meant that light availability would become the currency of the symbiotic relationships.

Indeed, some algae showed significant positive effects of Ag A on photosynthetic oxygen evolution under UV radiation. Over the whole range of nine microalgae and three UV radiation intensities, however, the effect of Ag A varied greatly, contradicting the initial hypothesis of universal effects. Consistent, positive effects of Ag A on oxygen evolution were only observed in *Synechococcus* sp. RCC1084 and *T*. *lutea*. This effect was with +41% to +921% greater in RCC1084 than in *T*. *lutea* (25–55%). In *Synechococcus* sp. RCC1084, the positive effect remained significant also under a combination of UV radiation and PAR, dependent on the radiation intensity. Oxygen evolution was increased by +41% at low and +21% at moderate light intensity, while no significant change was observed at high light intensity. This points to a decreasing importance of photons offered additionally by the fluorescence of Ag A with increasing PAR availability. Of the other algae, *Synechococcus* sp. RCC539, *C*. *elongatum* and *M*. *americana* showed no effect of Ag A addition under any UV radiation intensity. Furthermore, for *S*. *bacillaris* CCMP1333 and *R*. *baltica* any observed effect of Ag A on oxygen evolution was negative. While an increased availability of light energy due to the conversion from UV radiation to visible light may be one effect of Ag A, the great differences between species and even strains suggest more complex interactions of Ag A within the host/symbiont system than only a simple wavelength-transforming mechanism.

In addition, the presence of Ag A may also have acted like a sunscreen, protecting algae from negative effects of UV radiation in the “combined high” light treatment. While *Synechococcus* RCC1084 showed no adverse effects of UV radiation on oxygen evolution in presence of Ag A, compared to -29% in the controls, in *S*. *bacillaris* CCMP1333 the presence of Ag A alleviated the negative effect from -40% in the controls to -13%.

The two *Synechococcus* strains RCC1084 and CCMP1333 belong to the same pigment type 1, which is characterised by their phycobilisomes containing only allophycocyanin and C-phycocyanin [18]. And yet, despite the similar pigment composition, effects of Ag A on their photosynthetic efficiency under pure UV radiation varied greatly, highlighting the complex dynamics of Ag A and microalgae.

### Effects of Ag A: Pigment interactions

In a recent study [17], significant changes in algal fluorescence spectra were observed after Ag A additions, suggesting interactions between Ag A and various pigments of microalgae. However, while similar effects due to Ag A addition were observed in some pigment complexes, in other cases the chemical environment within the chloroplast appeared to alter the effects of Ag A on fluorescence.

Peter et al. [17] described that, in the presence of Ag A the fluorescence spectra of two *Synechococcus* strains belonging to pigment type 3, RCC539 and RCC791, were impacted most profoundly. The phycobilisomes of strains belonging to this pigment type are comprised of phycocyanin and two different variants of phycoerythrin [18]. While in the controls none of the algal pigments (phycocyanin, phycoerythrin, chl *a*) exhibited fluorescence peaks, in the presence of Ag A significant peaks attributable to phycoerythrin were observed. The authors hypothesised, that this drastic increase in fluorescence could indicate an uncoupling of phycobilisomes from the photosystems or even from the thylakoid membrane. The disruption in the energy funnelling pathway would then prevent the transfer of absorbed energy to the next molecule, thus reducing the photosynthetic efficiency. In line with this hypothesis, both strains exhibited significantly reduced oxygen evolution both under a combination of UV radiation and PAR and under sole PAR illumination. The reduction was substantial with at -61% to -80% in *Synechococcus* sp. RCC539 and -32% to -64% in RCC791.

Another remarkable observation of the study by Peter et al. [17] was the effect Ag A had on the fluorescence spectrum of *T*. *lutea*. In this case, chl *a* fluorescence greatly increased in the presence of Ag A, and its fluorescence maximum shifted from 685 nm in the controls to 676 nm. These two effects coincided with a significant, positive effects of Ag A on oxygen evolution observed in the present study, both under combined light treatments as well as under pure PAR. Interestingly, the greatest increase (31%) was observed at the lowest light intensity, indicating that the impact of Ag A on chl *a* depends on the capacity of other components of the photosynthetic apparatus such as the cytochrome b6f complex and D1 and D2 proteins.

In the two chlorophytes *C*. *elongatum* and *M*. *americana*, contrasting effects of Ag A on chl *a* fluorescence have previously been reported [17], with the amplitude of the peak decreased by -25% in *C*. *elongatum*, while it increased by +28% in *M*. *americana*. Since these two algae do not show consistent positive or negative effects of Ag A on gross oxygen evolution, the boost in photosynthetic efficiency observed in *T*. *lutea* may be connected to the shift in the chl *a* fluorescence peak observed in this alga and not to increased fluorescence intensity. Again, the observed differences in the interactions between chl *a* and Ag A highlight the diversity and complexity of algal photosynthetic apparatuses.

### Miscellaneous effects

*T*. *chuii* presented results markedly different from those of all other algae. Irrespective of the light treatment and even in darkness, the metabolic activity was consistently reduced by 75% to 193%. This resulted in a minimal gross oxygen loss even under maximum PAR intensity. Since this drastic negative effect of Ag A was observed irrespective of the light treatment, it is most probably not caused by changes in light availability due to Ag A or by an Ag A–pigment interaction. Given the importance of kinases for every aspect of cell function, their inhibition by Ag A, as assessed by Shengule et al. [4] could be the cause behind the observed reduction in metabolic activity and subsequent oxygen evolution. While the mechanisms behind these observations can only be speculated upon, one possible reason these effects were only observed in *T*. *chuii*, could be that marine chlorophytes employ kinases which are different from the other algae tested here.

Another interesting finding not connected to any effects of Ag A was the increased oxygen evolution in the *C*. *elongatum* controls, when exposed to a combination of UV radiation plus PAR compared to just PAR alone. While it suggests, that *C*. *elongatum* may be capable of harnessing UV radiation even in the absence of Ag A, this hypothesis requires further testing.

## Conclusions

Ag A exhibited a variety of effects on different microalgae. While in the case of *T*. *chuii* overall metabolic activity was greatly reduced under all lighting scenarios, *T*. *lutea* exhibited positive effects of Ag A on gross oxygen evolution under diverse light conditions. For *Synechococcus*, even strains belonging to the same pigment type reacted differently to the incubation with Ag A when exposed to UV radiation.

To date the means by which hosts acquire, assemble and maintain their symbiotic microbiomes remains mostly unresolved. Given the diversity of ways in which Ag A interacts with microalgae and the prevalence of Ag A-like molecules in a variety of marine hosts, their diverse role might be surprisingly universal: supporting microbial communities customised to the host.

## Supporting information

S1 Table Composition of phycobilisomes of the different *Synechococcus* pigment types [18]. (DOCX)

S2 Table Light intensity in the respective light treatments composed of UV radiation [mW m^-2^ s^-1^] and PAR [μmol photons m^-2^ s^-1^]. (DOCX)

S3 Table Analysed raw data of *Synechococcus* sp. RCC539. (DOCX)

S4 Table Analysed raw data of *Synechococcus* sp. RCC791. (DOCX)

S5 Table Analysed raw data of *Synechococcus* sp. RCC1084. (DOCX)

S6 Table Analysed raw data of *S*. *bacillaris* CCMP1333. (DOCX)

S7 Table Analysed raw data of *C*. *elongatum*. (DOCX)

S8 Table Analysed raw data of *M*. *Americana*. (DOCX)

S9 Table Analysed raw data of *T*. *chuii*. (DOCX)

S10 Table Analysed raw data of *T*. *lutea*. (DOCX)

S11 Table Analysed raw data of *R*. *baltica*. (DOCX)

S12 Table Difference in % O_2_ saturation per 10^6^ cells mL^-1^ after 90 min incubation in darkness. (DOCX)

S13 Table Difference in % O_2_ saturation per 10^3^ cells mL^-1^ after 90 min incubation in darkness. (DOCX)
