# Supplementary material for: Effects of sponge-derived Ageladine A on the photosynthesis of different microalgal species and strains
Source: PLoS One. 2020 Dec 31;15(12):e0244095. doi: 10.1371/journal.pone.0244095 (PMC7774917; doi:10.1371/journal.pone.0244095)
Supplement: S1 Table — (DOCX) [file pone.0244095.s001.docx]

|  | Allophycocyanin | C-Phycocyanin | R-Phycocyanin II or other | Phycoerythrin I | Phycoerythrin II |
| --- | --- | --- | --- | --- | --- |
| Pigment type 1 | x | x |  |  |  |
| Pigment type 2 | x | x | x | x | x |
| Pigment type 3 | x |  | x | x | x |
