# Supplementary material for: Effects of sponge-derived Ageladine A on the photosynthesis of different microalgal species and strains
Source: PLoS One. 2020 Dec 31;15(12):e0244095. doi: 10.1371/journal.pone.0244095 (PMC7774917; doi:10.1371/journal.pone.0244095)
Supplement: S2 Table — (DOCX) [file pone.0244095.s002.docx]

|  | PAR max | UV low | UV moderate | UV high | combined low | combined moderate | combined high |
| --- | --- | --- | --- | --- | --- | --- | --- |
| UV radiation  intensity | 31 | 250 | 630 | 1860 | 260 | 660 | 1930 |
| PAR  intensity | 47 | 0.65 | 1.6 | 4.7 | 6.7 | 14 | 51 |
