# Supplementary material for: Effects of sponge-derived Ageladine A on the photosynthesis of different microalgal species and strains
Source: PLoS One. 2020 Dec 31;15(12):e0244095. doi: 10.1371/journal.pone.0244095 (PMC7774917; doi:10.1371/journal.pone.0244095)
Supplement: S3 Table — (DOCX) [file pone.0244095.s003.docx]

|  |  |  | PAR max | darkness | UV low | combined low | UV moderate | combined moderate | UV high | combined high |
| --- | --- | --- | --- | --- | --- | --- | --- | --- | --- | --- |
| difference in O_2_ [%] | control | mean | 4.0 | -3.2 | -4.6 | -5.2 | -6.4 | 0.4 | -4.2 | 3.2 |
|  |  | sd | 1.0 | 0.4 | 0.5 | 1.3 | 0.9 | 0.9 | 0.8 | 0.8 |
|  | with Ag A | mean | -0.2 | -1.8 | -4.2 | -3.6 | -4.6 | -0.4 | -4.4 | -0.4 |
|  |  | sd | 0.8 | 0.4 | 0.5 | 0.4 | 0.5 | 0.5 | 1.5 | 0.5 |
| cell density compared to start cell density [%] | control |  | 89 | 87 | 87 | 90 | 84 | 72 | 69 | 91 |
|  | Ag A |  | 84 | 75 | 84 | 76 | 72 | 60 | 62 | 76 |
| difference in O_2_  [% (10^6^ cells mL^-1^)^-1^] | control | mean | 0.212 | -0.119 | -0.172 | -0.133 | -0.188 | 0.020 | -0.125 | 0.071 |
|  |  | sd | 0.053 | 0.017 | 0.021 | 0.033 | 0.026 | 0.045 | 0.025 | 0.019 |
|  | with Ag A | mean | -0.011 | -0.078 | -0.163 | -0.109 | -0.158 | -0.024 | -0.147 | -0.011 |
|  |  | sd | 0.047 | 0.019 | 0.017 | 0.017 | 0.019 | 0.033 | 0.051 | 0.014 |
| gross difference in O_2_ [% (10^6^ cells mL^-1^)^-1^] | control | mean | 0.331 |  | -0.053 | -0.014 | -0.069 | 0.140 | -0.006 | 0.191 |
|  |  | sd | 0.055 |  | 0.026 | 0.037 | 0.031 | 0.048 | 0.030 | 0.025 |
|  | with Ag A | mean | 0.067 |  | -0.085 | -0.031 | -0.080 | 0.054 | -0.068 | 0.068 |
|  |  | sd | 0.051 |  | 0.026 | 0.026 | 0.027 | 0.038 | 0.054 | 0.024 |
