# Supplementary material for: Effects of sponge-derived Ageladine A on the photosynthesis of different microalgal species and strains
Source: PLoS One. 2020 Dec 31;15(12):e0244095. doi: 10.1371/journal.pone.0244095 (PMC7774917; doi:10.1371/journal.pone.0244095)
Supplement: S4 Table — (DOCX) [file pone.0244095.s004.docx]

|  |  |  | PAR max | darkness | UV low | combined low | UV moderate | combined moderate | UV high | combined high |
| --- | --- | --- | --- | --- | --- | --- | --- | --- | --- | --- |
| difference in O_2_ [%] | control | mean | 9.0 | -10.2 | -9.2 | -5.4 | -14.8 | -1.0 | -7.2 | 6.6 |
|  |  | sd | 0.7 | 0.4 | 0.4 | 1.1 | 1.3 | 0.7 | 1.8 | 0.9 |
|  | with Ag A | mean | 2.4 | -6.4 | -5.4 | -4.2 | -6.6 | -0.8 | -5.0 | -1.0 |
|  |  | sd | 0.5 | 0.5 | 0.5 | 0.4 | 0.5 | 0.8 | 0.7 | 0.7 |
| cell density compared to start cell density [%] | control |  | 111 | 116 | 109 | 111 | 149 | 103 | 107 | 108 |
|  | Ag A |  | 107 | 104 | 100 | 99 | 125 | 100 | 111 | 123 |
| difference in O_2_  [% (10^6^ cells mL^-1^)^-1^] | control | mean | 0.127 | -0.085 | -0.081 | -0.046 | -0.115 | -0.011 | -0.086 | 0.058 |
|  |  | sd | 0.010 | 0.004 | 0.004 | 0.010 | 0.010 | 0.008 | 0.021 | 0.008 |
|  | with Ag A | mean | 0.035 | -0.060 | -0.052 | -0.040 | -0.061 | -0.009 | -0.058 | -0.008 |
|  |  | sd | 0.008 | 0.005 | 0.005 | 0.004 | 0.005 | 0.010 | 0.008 | 0.005 |
| gross difference in O_2_ [% (10^6^ cells mL^-1^)^-1^] | control | mean | 0.212 |  | 0.005 | 0.039 | -0.030 | 0.074 | -0.001 | 0.144 |
|  |  | sd | 0.011 |  | 0.005 | 0.010 | 0.011 | 0.009 | 0.022 | 0.009 |
|  | with Ag A | mean | 0.095 |  | 0.008 | 0.019 | -0.001 | 0.050 | 0.002 | 0.052 |
|  |  | sd | 0.010 |  | 0.007 | 0.007 | 0.007 | 0.011 | 0.010 | 0.007 |
