# Supplementary material for: Effects of sponge-derived Ageladine A on the photosynthesis of different microalgal species and strains
Source: PLoS One. 2020 Dec 31;15(12):e0244095. doi: 10.1371/journal.pone.0244095 (PMC7774917; doi:10.1371/journal.pone.0244095)
Supplement: S5 Table — (DOCX) [file pone.0244095.s005.docx]

|  |  |  | PAR max | darkness | UV low | combined low | UV moderate | combined moderate | UV high | combined high |
| --- | --- | --- | --- | --- | --- | --- | --- | --- | --- | --- |
| difference in O_2_ [%] | control | mean | 18.0 | -16.6 | -9.2 | -6.6 | -15.0 | -0.6 | -14.2 | 4.2 |
|  |  | sd | 3.5 | 0.9 | 0.4 | 1.7 | 1.2 | 0.5 | 1.8 | 0.4 |
|  | with Ag A | mean | 9.4 | -6.4 | -6.0 | -2.4 | -8.0 | 0.4 | -8.4 | 4.6 |
|  |  | sd | 1.7 | 0.9 | 0.7 | 0.9 | 1.0 | 0.5 | 0.5 | 0.5 |
| cell density compared to start cell density [%] | control |  | 115 | 105 | 108 | 109 | 106 | 109 | 116 | 100 |
|  | Ag A |  | 107 | 101 | 101 | 103 | 104 | 107 | 112 | 102 |
| difference in O_2_  [% (10^6^ cells mL^-1^)^-1^] | control | mean | 0.305 | -0.297 | -0.168 | -0.118 | -0.282 | -0.033 | -0.236 | 0.131 |
|  |  | sd | 0.059 | 0.016 | 0.008 | 0.030 | 0.023 | 0.030 | 0.030 | 0.014 |
|  | with Ag A | mean | 0.172 | -0.119 | -0.117 | -0.045 | -0.154 | 0.023 | -0.145 | 0.140 |
|  |  | sd | 0.031 | 0.017 | 0.014 | 0.017 | 0.019 | 0.031 | 0.009 | 0.017 |
| gross difference in O_2_ [% (10^6^ cells mL^-1^)^-1^] | control | mean | 0.602 |  | 0.128 | 0.179 | 0.014 | 0.264 | 0.061 | 0.428 |
|  |  | sd | 0.061 |  | 0.018 | 0.034 | 0.028 | 0.034 | 0.034 | 0.021 |
|  | with Ag A | mean | 0.469 |  | 0.180 | 0.252 | 0.143 | 0.320 | 0.152 | 0.437 |
|  |  | sd | 0.035 |  | 0.022 | 0.024 | 0.025 | 0.035 | 0.019 | 0.024 |
