# Supplementary material for: Effects of sponge-derived Ageladine A on the photosynthesis of different microalgal species and strains
Source: PLoS One. 2020 Dec 31;15(12):e0244095. doi: 10.1371/journal.pone.0244095 (PMC7774917; doi:10.1371/journal.pone.0244095)
Supplement: S6 Table — (DOCX) [file pone.0244095.s006.docx]

|  |  |  | PAR max | darkness | UV low | combined low | UV moderate | combined moderate | UV high | combined high |
| --- | --- | --- | --- | --- | --- | --- | --- | --- | --- | --- |
| difference in O_2_ [%] | control | mean | 19.8 | -21.8 | -26.2 | -13.8 | -22.0 | 1.4 | -13.8 | 5.4 |
|  |  | sd | 2.3 | 0.8 | 1.1 | 1.8 | 2.8 | 3.0 | 1.9 | 1.8 |
|  | with Ag A | mean | 12.2 | -11.2 | -14.2 | -3.6 | -11.6 | 3.2 | -6.8 | 9.8 |
|  |  | sd | 1.3 | 0.4 | 0.4 | 0.9 | 1.1 | 0.4 | 1.1 | 0.4 |
| cell density compared to start cell density [%] | control |  | 111 | 109 | 139 | 111 | 102 | 127 | 104 | 145 |
|  | Ag A |  | 119 | 99 | 117 | 102 | 92 | 114 | 92 | 131 |
| difference in O_2_  [% (10^6^ cells mL^-1^)^-1^] | control | mean | 0.559 | -0.533 | -0.487 | -0.340 | -0.587 | 0.033 | -0.348 | 0.121 |
|  |  | sd | 0.064 | 0.020 | 0.020 | 0.044 | 0.075 | 0.069 | 0.048 | 0.041 |
|  | with Ag A | mean | 0.324 | -0.303 | -0.313 | -0.097 | -0.344 | 0.084 | -0.195 | 0.242 |
|  |  | sd | 0.035 | 0.012 | 0.010 | 0.024 | 0.034 | 0.012 | 0.031 | 0.011 |
| gross difference in O_2_ [% (10^6^ cells mL^-1^)^-1^] | control | mean | 1.092 |  | 0.046 | 0.193 | -0.054 | 0.566 | 0.185 | 0.654 |
|  |  | sd | 0.068 |  | 0.029 | 0.049 | 0.078 | 0.072 | 0.053 | 0.045 |
|  | with Ag A | mean | 0.626 |  | -0.011 | 0.206 | -0.042 | 0.386 | 0.107 | 0.545 |
|  |  | sd | 0.037 |  | 0.016 | 0.027 | 0.036 | 0.017 | 0.034 | 0.016 |
