# Supplementary material for: Effects of sponge-derived Ageladine A on the photosynthesis of different microalgal species and strains
Source: PLoS One. 2020 Dec 31;15(12):e0244095. doi: 10.1371/journal.pone.0244095 (PMC7774917; doi:10.1371/journal.pone.0244095)
Supplement: S7 Table — (DOCX) [file pone.0244095.s007.docx]

|  |  |  | PAR max | darkness | UV low | combined low | UV moderate | combined moderate | UV high | combined high |
| --- | --- | --- | --- | --- | --- | --- | --- | --- | --- | --- |
| difference in O_2_ [%] | control | mean | 12.6 | -13.4 | -3.8 | -6.6 | -7.2 | 2.2 | -1.8 | 26.8 |
|  |  | sd | 1.5 | 2.7 | 1.3 | 1.3 | 0.8 | 0.8 | 2.9 | 5.0 |
|  | with Ag A | mean | 15.6 | -9.8 | -3.0 | -6.2 | -8.4 | 5.0 | -2.0 | 23.6 |
|  |  | sd | 2.1 | 1.1 | 0.0 | 1.3 | 2.3 | 2.9 | 1.2 | 2.3 |
| cell density compared to start cell density [%] | control |  | 76 | 67 | 115 | 98 | 86 | 85 | 76 | 53 |
|  | Ag A |  | 68 | 39 | 101 | 86 | 86 | 54 | 83 | 60 |
| difference in O_2_  [% (10^3^ cells mL^-1^)^-1^] | control | mean | 0.075 | -0.105 | -0.021 | -0.033 | -0.032 | 0.012 | -0.011 | 0.175 |
|  |  | sd | 0.009 | 0.021 | 0.007 | 0.007 | 0.004 | 0.005 | 0.017 | 0.032 |
|  | with Ag A | mean | 0.103 | -0.131 | -0.019 | -0.035 | -0.038 | 0.044 | -0.011 | 0.135 |
|  |  | sd | 0.014 | 0.015 | 0.000 | 0.007 | 0.010 | 0.026 | 0.007 | 0.013 |
| gross difference in O_2_ [% (10^3^ cells mL^-1^)^-1^] | control | mean | 0.180 |  | 0.083 | 0.072 | 0.072 | 0.117 | 0.094 | 0.280 |
|  |  | sd | 0.023 |  | 0.022 | 0.022 | 0.021 | 0.022 | 0.027 | 0.039 |
|  | with Ag A | mean | 0.234 |  | 0.111 | 0.096 | 0.093 | 0.175 | 0.120 | 0.266 |
|  |  | sd | 0.020 |  | 0.015 | 0.016 | 0.018 | 0.029 | 0.016 | 0.020 |
