# Supplementary material for: Effects of sponge-derived Ageladine A on the photosynthesis of different microalgal species and strains
Source: PLoS One. 2020 Dec 31;15(12):e0244095. doi: 10.1371/journal.pone.0244095 (PMC7774917; doi:10.1371/journal.pone.0244095)
Supplement: S8 Table — (DOCX) [file pone.0244095.s008.docx]

|  |  |  | PAR max | darkness | UV low | combined low | UV moderate | combined moderate | UV high | combined high |
| --- | --- | --- | --- | --- | --- | --- | --- | --- | --- | --- |
| difference in O_2_ [%] | control | mean | 13.6 | -16.6 | -17.2 | -17.6 | -20.0 | 0.0 | -9.6 | 7.0 |
|  |  | sd | 1.7 | 1.7 | 2.6 | 3.0 | 1.9 | 4.3 | 2.4 | 1.9 |
|  | with Ag A | mean | 15.4 | -16.0 | -14.0 | -12.8 | -16.0 | 2.0 | -9.2 | 13.4 |
|  |  | sd | 6.1 | 1.2 | 2.0 | 1.8 | 2.8 | 1.2 | 2.9 | 3.2 |
| cell density compared to start cell density [%] | control |  | 79 | 59 | 114 | 98 | 105 | 97 | 100 | 98 |
|  | Ag A |  | 67 | 66 | 92 | 117 | 88 | 112 | 82 | 104 |
| difference in O_2_  [% (10^3^ cells mL^-1^)^-1^] | control | mean | 3.676 | -2.243 | -1.575 | -2.005 | -2.740 | 0.000 | -1.455 | 1.282 |
|  |  | sd | 0.452 | 0.226 | 0.237 | 0.347 | 0.256 | 0.618 | 0.365 | 0.343 |
|  | with Ag A | mean | 4.968 | -1.951 | -1.587 | -1.228 | -2.606 | 0.250 | -1.685 | 2.302 |
|  |  | sd | 1.970 | 0.149 | 0.227 | 0.172 | 0.461 | 0.153 | 0.540 | 0.551 |
| gross difference in O_2_ [% (10^3^ cells mL^-1^)^-1^] | control | mean | 5.919 |  | 0.668 | 0.239 | -0.496 | 2.243 | 0.789 | 3.525 |
|  |  | sd | 0.506 |  | 0.328 | 0.414 | 0.342 | 0.658 | 0.429 | 0.411 |
|  | with Ag A | mean | 6.919 |  | 0.364 | 0.723 | -0.655 | 2.201 | 0.266 | 4.254 |
|  |  | sd | 1.976 |  | 0.272 | 0.228 | 0.484 | 0.214 | 0.560 | 0.571 |
