# Supplementary material for: Effects of sponge-derived Ageladine A on the photosynthesis of different microalgal species and strains
Source: PLoS One. 2020 Dec 31;15(12):e0244095. doi: 10.1371/journal.pone.0244095 (PMC7774917; doi:10.1371/journal.pone.0244095)
Supplement: S9 Table — (DOCX) [file pone.0244095.s009.docx]

|  |  |  | PAR max | darkness | UV low | combined low | UV moderate | combined moderate | UV high | combined high |
| --- | --- | --- | --- | --- | --- | --- | --- | --- | --- | --- |
| difference in O_2_ [%] | control | mean | 10.4 | -14.2 | -13.2 | -13.8 | -12.2 | 1.6 | -6.4 | 9.2 |
|  |  | sd | 1.7 | 0.8 | 1.5 | 1.9 | 2.0 | 1.1 | 1.1 | 2.6 |
|  | with Ag A | mean | -1.6 | -1.2 | -2.0 | -2.0 | -4.6 | -0.4 | -4.0 | -2.6 |
|  |  | sd | 1.1 | 0.8 | 1.0 | 0.7 | 1.3 | 1.1 | 0.7 | 0.9 |
| cell density compared to start cell density [%] | control |  | 120 | 137 | 131 | 102 | 73 | 89 | 106 | 162 |
|  | Ag A |  | 195 | 172 | 161 | 185 | 192 | 170 | 170 | 179 |
| difference in O_2_  [% (10^3^ cells mL^-1^)^-1^] | control | mean | 0.200 | -0.238 | -0.167 | -0.265 | -0.400 | 0.040 | -0.180 | 0.127 |
|  |  | sd | 0.032 | 0.014 | 0.019 | 0.037 | 0.067 | 0.028 | 0.032 | 0.036 |
|  | with Ag A | mean | -0.019 | -0.016 | -0.021 | -0.021 | -0.057 | -0.005 | -0.070 | -0.032 |
|  |  | sd | 0.013 | 0.011 | 0.010 | 0.008 | 0.017 | 0.015 | 0.012 | 0.011 |
| gross difference in O_2_ [% (10^3^ cells mL^-1^)^-1^] | control | mean | 0.438 |  | 0.071 | -0.027 | -0.162 | 0.278 | 0.058 | 0.365 |
|  |  | sd | 0.035 |  | 0.023 | 0.040 | 0.069 | 0.032 | 0.035 | 0.038 |
|  | with Ag A | mean | -0.003 |  | -0.005 | -0.005 | -0.041 | 0.011 | -0.054 | -0.016 |
|  |  | sd | 0.018 |  | 0.015 | 0.014 | 0.020 | 0.019 | 0.017 | 0.016 |
