# Supplementary material for: Effects of sponge-derived Ageladine A on the photosynthesis of different microalgal species and strains
Source: PLoS One. 2020 Dec 31;15(12):e0244095. doi: 10.1371/journal.pone.0244095 (PMC7774917; doi:10.1371/journal.pone.0244095)
Supplement: S10 Table — (DOCX) [file pone.0244095.s010.docx]

|  |  |  | PAR max | darkness | UV low | combined low | UV moderate | combined moderate | UV high | combined high |
| --- | --- | --- | --- | --- | --- | --- | --- | --- | --- | --- |
| difference in O_2_ [%] | control | mean | 13.4 | -17.4 | -18.0 | -3.8 | -10.0 | 1.2 | -18.0 | 10.6 |
|  |  | sd | 2.3 | 2.2 | 1.2 | 3.4 | 1.0 | 3.4 | 2.5 | 2.1 |
|  | with Ag A | mean | 14.2 | -14.0 | -16.2 | -1.8 | -11.6 | 0.6 | -12.6 | 15.6 |
|  |  | sd | 2.3 | 0.7 | 1.5 | 4.1 | 1.1 | 1.8 | 2.3 | 3.8 |
| cell density compared to start cell density [%] | control |  | 86 | 46 | 105 | 110 | 89 | 97 | 91 | 92 |
|  | Ag A |  | 83 | 30 | 100 | 112 | 89 | 112 | 93 | 97 |
| difference in O_2_  [% (10^3^ cells mL^-1^)^-1^] | control | mean | 0.033 | -0.081 | -0.030 | -0.007 | -0.018 | 0.002 | -0.031 | 0.021 |
|  |  | sd | 0.006 | 0.010 | 0.002 | 0.006 | 0.002 | 0.006 | 0.004 | 0.004 |
|  | with Ag A | mean | 0.036 | -0.101 | -0.028 | -0.003 | -0.020 | 0.001 | -0.021 | 0.029 |
|  |  | sd | 0.006 | 0.005 | 0.003 | 0.007 | 0.002 | 0.003 | 0.004 | 0.007 |
| gross difference in O_2_ [% (10^3^ cells mL^-1^)^-1^] | control | mean | 0.115 |  | 0.051 | 0.075 | 0.064 | 0.084 | 0.051 | 0.102 |
|  |  | sd | 0.012 |  | 0.010 | 0.012 | 0.010 | 0.012 | 0.011 | 0.011 |
|  | with Ag A | mean | 0.137 |  | 0.072 | 0.098 | 0.080 | 0.102 | 0.079 | 0.129 |
|  |  | sd | 0.008 |  | 0.006 | 0.009 | 0.005 | 0.006 | 0.006 | 0.009 |
