# Supplementary material for: Effects of sponge-derived Ageladine A on the photosynthesis of different microalgal species and strains
Source: PLoS One. 2020 Dec 31;15(12):e0244095. doi: 10.1371/journal.pone.0244095 (PMC7774917; doi:10.1371/journal.pone.0244095)
Supplement: S11 Table — (DOCX) [file pone.0244095.s011.docx]

|  |  |  | PAR max | darkness | UV low | combined low | UV moderate | combined moderate | UV high | combined high |
| --- | --- | --- | --- | --- | --- | --- | --- | --- | --- | --- |
| difference in O_2_ [%] | control | mean | 18.4 | -32.6 | -15.6 | -18.6 | -22.4 | 7.0 | -7.4 | 17.2 |
|  |  | sd | 1.5 | 1.3 | 1.9 | 2.1 | 1.1 | 2.0 | 0.5 | 7.2 |
|  | with Ag A | mean | 12.4 | -23.4 | -12.4 | -16.4 | -23.6 | 4.0 | -9.6 | 9.4 |
|  |  | sd | 2.7 | 1.5 | 2.1 | 0.9 | 1.8 | 1.6 | 0.9 | 1.5 |
| cell density compared to start cell density [%] | control |  | 106 | 127 | 92 | 99 | 121 | 108 | 106 | 117 |
|  | Ag A |  | 106 | 105 | 96 | 93 | 115 | 99 | 106 | 102 |
| difference in O_2_  [% (10^3^ cells mL^-1^)^-1^] | control | mean | 0.081 | -0.126 | -0.074 | -0.077 | -0.097 | 0.030 | -0.031 | 0.072 |
|  |  | sd | 0.007 | 0.005 | 0.009 | 0.009 | 0.005 | 0.009 | 0.002 | 0.030 |
|  | with Ag A | mean | 0.054 | -0.110 | -0.056 | -0.073 | -0.108 | 0.019 | -0.041 | 0.045 |
|  |  | sd | 0.012 | 0.007 | 0.009 | 0.004 | 0.008 | 0.007 | 0.004 | 0.007 |
| gross difference in O_2_ [% (10^3^ cells mL^-1^)^-1^] | control | mean | 0.207 |  | 0.053 | 0.049 | 0.029 | 0.157 | 0.095 | 0.199 |
|  |  | sd | 0.008 |  | 0.011 | 0.010 | 0.007 | 0.010 | 0.006 | 0.031 |
|  | with Ag A | mean | 0.164 |  | 0.053 | 0.037 | 0.002 | 0.129 | 0.069 | 0.155 |
|  |  | sd | 0.014 |  | 0.012 | 0.008 | 0.011 | 0.010 | 0.008 | 0.010 |
