# Supplementary material for: Effects of sponge-derived Ageladine A on the photosynthesis of different microalgal species and strains
Source: PLoS One. 2020 Dec 31;15(12):e0244095. doi: 10.1371/journal.pone.0244095 (PMC7774917; doi:10.1371/journal.pone.0244095)
Supplement: S12 Table — (DOCX) [file pone.0244095.s012.docx]

|  | *Synechococcus* sp. RCC539 | | *Synechococcus* sp. RCC791 | | *Synechococcus* sp. RCC1084 | | *S. bacillaris* CCMP1333 | |
| --- | --- | --- | --- | --- | --- | --- | --- | --- |
|  | mean | sd | mean | sd | mean | sd | mean | sd |
| control | -0.119 | 0.017 | -0.085 | 0.004 | -0.297 | 0.016 | -0.533 | 0.020 |
| Ag A | -0.078 | 0.019 | -0.060 | 0.005 | -0.119 | 0.017 | -0.303 | 0.012 |
