# Supplementary material for: Effects of sponge-derived Ageladine A on the photosynthesis of different microalgal species and strains
Source: PLoS One. 2020 Dec 31;15(12):e0244095. doi: 10.1371/journal.pone.0244095 (PMC7774917; doi:10.1371/journal.pone.0244095)
Supplement: S13 Table — (DOCX) [file pone.0244095.s013.docx]

|  | *C. elongatum* | | *M. americana* | | *T. chuii* | | *T. lutea* | | *R. baltica* | |
| --- | --- | --- | --- | --- | --- | --- | --- | --- | --- | --- |
|  | mean | sd | mean | sd | mean | sd | mean | sd | mean | sd |
| control | -0.105 | 0.021 | -2.243 | 0.226 | -0.238 | 0.014 | -0.081 | 0.010 | -0.126 | 0.005 |
| Ag A | -0.131 | 0.015 | -1.951 | 0.149 | -0.016 | 0.011 | -0.101 | 0.005 | -0.110 | 0.007 |
